# Comparative Evaluation of Microhardness and Polymerization Shrinkage in Residual Zirconia Reinforced Provisional Restorations: An In Vitro Study

**DOI:** 10.7759/cureus.64971

**Published:** 2024-07-20

**Authors:** Himaja Naina, Vidyashree V Nandini, Dilip M Kumar, Russia Marimuthu, Shiney Boruah, Naveen S Raj

**Affiliations:** 1 Prosthodontics, Sri Ramaswamy Memorial (SRM) Kattankulathur Dental College and Hospital, Chengalpattu, IND

**Keywords:** milling dust, cad-cam residual, green dentistry, cad-cam waste, residual zirconia

## Abstract

Aim

The aim of this study is to evaluate and compare the microhardness and polymerization shrinkage of polymethyl methacrylate reinforced with residual zirconia.

Materials and methods

A total of 360 resin samples were fabricated, with dimensions of 12 mm × 12 mm × 3 mm and 12 mm × 8 mm × 6 mm. Zirconia dust (40% by weight) was added to 180 of these samples. The study included four groups: Group A (autopolymerizing acrylic resin), Group H (heat-polymerizing acrylic resin), Group ZA (autopolymerizing acrylic resin with zirconia dust), and Group ZH (heat-polymerizing acrylic resin with zirconia dust). Each group consisted of 90 samples, with 45 samples used for evaluating microhardness and 45 samples for assessing polymerization shrinkage.

Results

Group ZH exhibited the highest microhardness at 6.06 ± 0.31 GPa. It also recorded the lowest shrinkage values, measuring 52.11 ± 3.21 mm³. Tukey’s honestly significant difference test revealed that microhardness was significantly higher in Group ZA (4.53 ± 0.29 GPa) compared to Group A (3.51 ± 0.25 GPa). However, Group H (5.42 ± 0.26 GPa) demonstrated greater hardness than Group ZA. Regarding shrinkage, the addition of zirconia dust resulted in reduced values, with Group ZA (73.93 ± 3.55 mm³) showing less shrinkage compared to Group A (91.9 ± 6.38 mm³). Similarly, Group ZH (52.11 ± 3.21 mm³) had lower shrinkage than Group H (66.71 ± 5.97 mm³). Group A exhibited the highest shrinkage among all the groups.

Conclusion

Within the limitations of this study, it can be concluded that there is an increase in hardness and a decrease in shrinkage values of the resin with the addition of zirconia dust in heat and autopolymerizing acrylic resin used for the fabrication of provisional restorations. Zirconia-incorporated heat-activated resin showed superior microhardness and decreased shrinkage values. Although the addition of residual zirconia to autopolymerized samples demonstrated better hardness, it was observed that pure heat-polymerized samples showed greater hardness. Reusing computer-aided design and computer-aided manufacturing powder waste can cut down on economic losses and aid in environmental sustainability.

## Introduction

The rise in cosmetic demand has led to advancements in the field of all ceramic restorations. This has resulted in new processing techniques to process all-ceramic materials, like heat press, slip casting, and additive and subtractive manufacturing. The introduction of these new processes has made manufacturing easier and more accessible. As zirconia is difficult to process, methods like subtractive technique, computer-aided design and computer-aided manufacturing (CAD/CAM), and additive techniques like three-dimensional printing have gained momentum. Pre-fabricated zirconia blanks are available in various shades and are milled into different dental prostheses like crowns, inlays and onlays, and veneers, making them very convenient and accessible. Consequently, the subtractive method of processing compact zirconia has resulted in the production of large amounts of zirconia dust (30%) and block residuals (80%), which causes economic and environmental losses. Furthermore, containing this waste would be eco-friendly and would also help economically [1].

Polymethyl methacrylate has been the most used material for the fabrication of provisional restorations, especially for long-term use. Temporary crowns/fixed dental prosthesis is an important step in fixed prosthodontic treatment. It helps to maintain tooth function after tooth preparation and the gingival contour. One of the main drawbacks of polymethyl methacrylate is its polymerization shrinkage. It should possess good mechanical strength and hardness so that it can be used for long-term prosthesis and withstand occlusal forces, especially in the posterior regions [2]. Thus, the study was begun with the purpose of evaluating hardness and polymerization shrinkage with the incorporation of zirconia dust residuals obtained from CAD/CAM milling, in provisional restoration materials.

The aim of the study was to evaluate the incorporation of zirconia residual powder in polymethyl methacrylate to fabricate provisional restorations and to evaluate the mechanical properties like microhardness and polymerization shrinkage of zirconia residual reinforced polymethyl methacrylate. This study began with two null hypotheses, the first being that the microhardness of pure polymethyl methacrylate and residual zirconia-incorporated polymethyl methacrylate would be the same, and the second was that the polymerization shrinkage of the polymethyl methacrylate and residual zirconia-incorporated polymethyl methacrylate would be the same.

## Materials and methods

**Figure 1:**
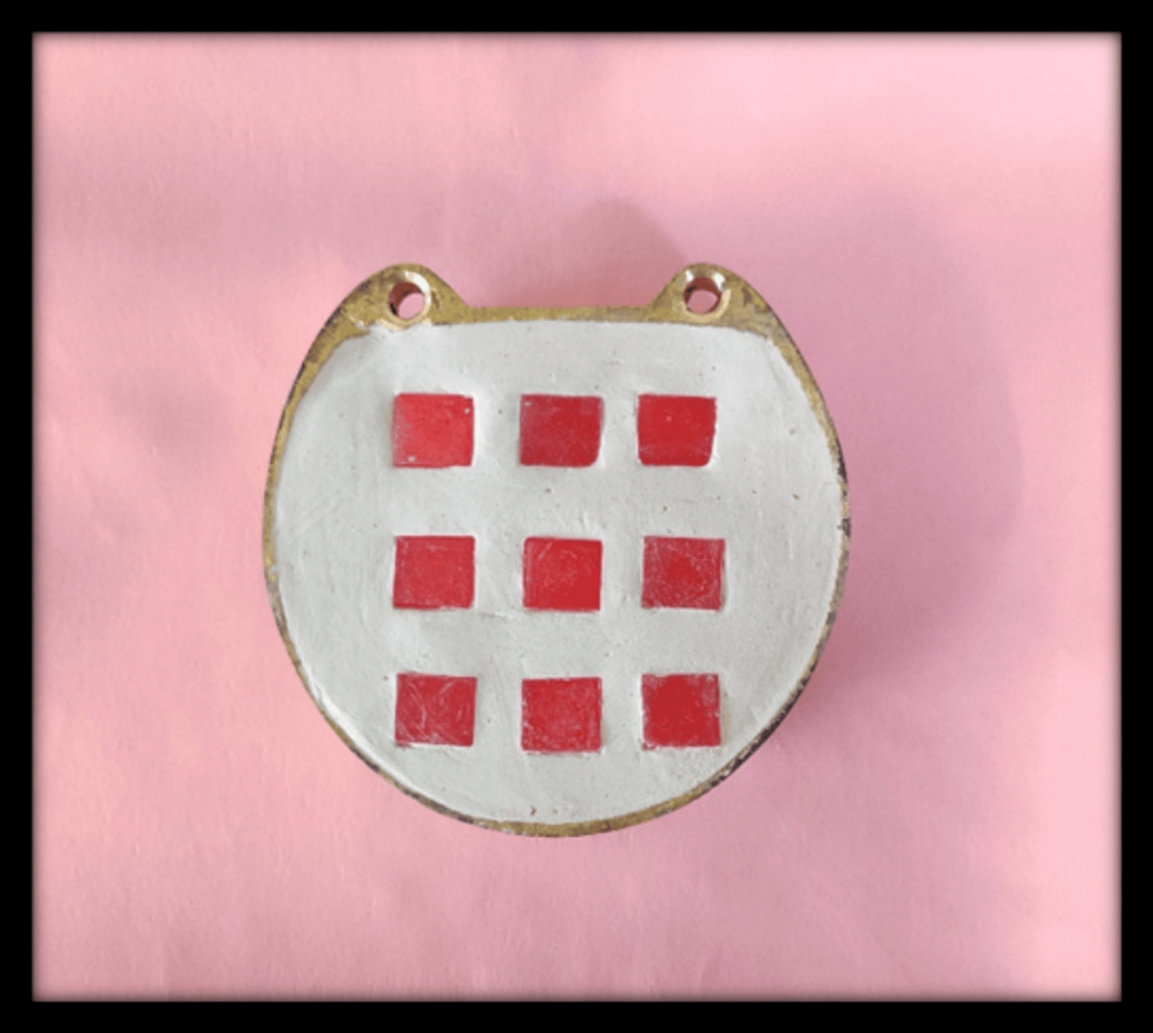
Wax patterns of samples for testing microhardness

**Figure 2:**
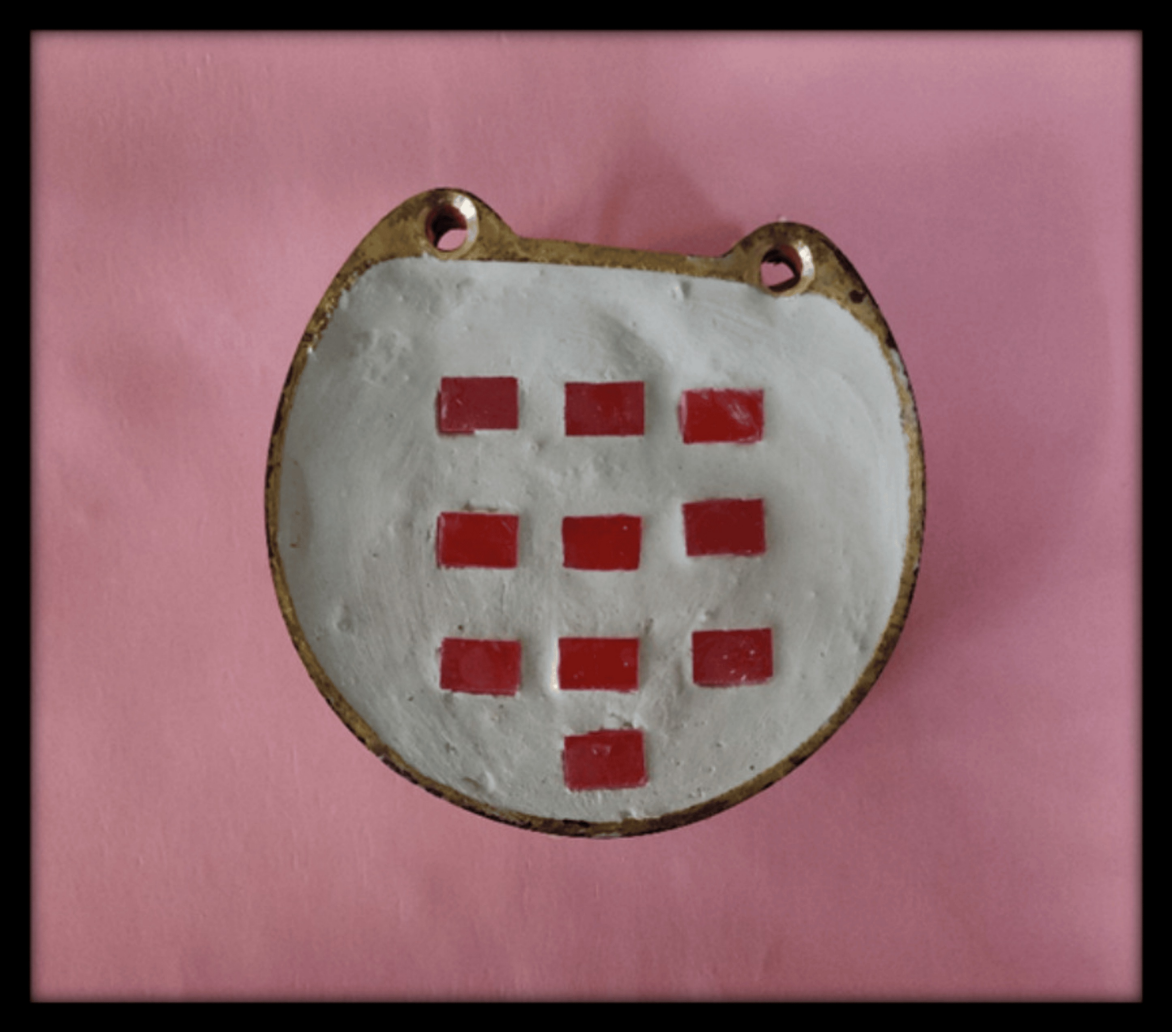
Wax patterns of samples for testing polymerization shrinkage

Rectangular stainless steel dies of dimensions 12 mm × 12 mm × 3 mm (Figure 1) and 12 mm × 8 mm × 6 mm (Figure 2) were fabricated for microhardness and polymerization shrinkage, respectively. Two molds were made of vinyl polysiloxane (A-silicone) duplicating material (Elite Double 22 Fast, Zhermack, Italy) using stainless steel dies. There were four groups in this study: Group A - autopolymerized resin samples, Group ZA - autopolymerized resin samples with 40% zirconia dust, Group H - heat polymerized resin samples, and Group ZH - heat polymerized resin samples with 40% zirconia dust.

For the preparation of the samples, 40 grams of zirconia dust (NexxZr T - Sagemax, Ivoclar Vivadent CAD-CAM Block, USA) and 60 grams of autopolymerizing monomer powder (DPI, Mumbai, India) were weighed and mixed with liquid in a ratio of 1 g/0.5 ml as per the manufacturer’s instructions [3]. These samples were retrieved from the molds, trimmed, and polished.

As for the heat-polymerized acrylic resin samples, wax patterns were fabricated in the mold created using the dies. These wax patterns were flasked and dewaxed at 100 ºC for four minutes. Similar to the autopolymerizing samples, zirconia dust and heat cure monomer (DPI, Mumbai, India) were weighed and mixed with liquid and then packed using the conventional packing method. Finally, processing of these samples was done in the acrylizer for two hours at 74 ºC, and the temperature was gradually increased to 100 ºC and processed for one hour [4]. All samples were then trimmed and polished. Thus, a total of 360 samples with two dimensions were fabricated (180 samples of each dimension) with 90 samples in Group A, 90 samples in Group ZA, 90 samples in Group H, and 90 samples in Group ZH. Out of the 90 samples in each group, 45 were for microhardness testing and the other 45 for polymerization shrinkage.

Microhardness and polymerization shrinkage evaluation

The samples with dimensions of 12 mm × 12 mm × 3 mm were fabricated for the evaluation of microhardness using the nanoindentation test. The nanoindenter has a sample base to which the samples were placed, an indenter shaft to which the indenter tip is attached, and a loading cell. The nanoindenter is computer-operated and records the load and displacement simultaneously. During the loading and unloading procedure, a precise trace of the indenter load and the related indenter displacement can be produced [5].

Samples for polymerization shrinkage were fabricated with dimensions of 12 mm × 8 mm × 6 mm. The shrinkage of the samples was calculated by using a vision measuring machine (VMM). The VMM is a non-contact calibration system that is powered by a computer and helps to assess the mechanical properties of objects with smaller dimensions [6]. VMM was used to ascertain the samples’ length, width, and thickness. Along with resin samples, the rectangular stainless steel die was also measured for length, breadth, and thickness. The volume of the samples was determined by using the following formula: V = l × b × t.

The volume of the die that was prepared was used as a reference to evaluate the shrinkage of the samples by determining the difference in the volume of the die and sample. The data obtained for microhardness and polymerization shrinkage were analyzed using IBM SPSS Statistics for Windows, Version 27.0 (Released 2020; IBM Corp., Armonk, NY, USA). Using the Shapiro-Wilk test, the data was assessed for normality. A one-way ANOVA was employed to analyze the microhardness and polymerization shrinkage. Multiple pairwise comparisons were then made using Tukey’s honestly significant difference (HSD) test (post-hoc analysis) (α = 0.05).

## Results

The mean SD of the microhardness values of the four groups is depicted in Table 1. The highest amount of hardness value was recorded in Group ZH (6.06 ± 0.31 GPa), and the lowest value was recorded in Group A (3.51 ± 0.25 GPa). Moreover, it was observed that Group H recorded higher hardness values (5.42 ± 0.26 GPa) than Group ZA (4.53 ± 0.29 GPa). Table 2 depicts the mean SD of polymerization shrinkage. It was seen that the maximum amount of shrinkage was in Group A (91.9 ± 6.38 mm^3^), and the least shrinkage among the groups was recorded in Group ZH (52.11 ± 3.21 mm^3^).

**Table 1:** Mean SD of microhardness of the four groups

Microhardness	Mean ± SD (GPa)
Group A	3.51 ± 0.25
Group H	5.42 ± 0.26
Group ZA	4.53 ± 0.29
Group ZH	6.06 ± 0.31

**Table 2:** Mean SD of polymerization shrinkage of the four groups

Shrinkage	Mean ± SD (mm^3^)
Group A	91.9 ± 6.38
Group H	66.71 ± 5.97
Group ZA	73.93 ± 3.55
Group ZH	52.11 ± 3.21

A one-way ANOVA test showed statistical significance at p < 0.05 for microhardness and polymerization shrinkage. Table 3 shows the results of multiple comparisons of microhardness using Tukey’s HSD test. The mean difference in microhardness between Group A and Group ZH was -3.72889 GPa, and between Group ZA and Group ZH was -2.71111 GPa, with a standard error of 0.83. The results of Tukey’s test were statistically significant (p < 0.05) for the comparison of the microhardness of Group ZH with Groups A and ZA.

**Table 3:** Multiple comparisons of microhardness among the groups using Tukey’s HSD test * The mean difference is significant at the 0.05 level. HSD, honestly significant difference

(I) Group	(J) Group	Mean difference (I-J)	Standard error	p-value	95% CI	
					Lower bound	Upper bound
Group A	Group H	-1.91111	0.8339	0.104	-4.074	0.2518
	Group ZA	-1.01778	0.8339	0.615	-3.1807	1.1451
	Group ZH	-3.72889^*^	0.8339	0	-5.8918	-1.566
Group H	Group A	1.91111	0.8339	0.104	-0.2518	4.074
	Group ZA	0.89333	0.8339	0.707	-1.2696	3.0563
	Group ZH	-1.81778	0.8339	0.133	-3.9807	0.3451
Group ZA	Group A	1.01778	0.8339	0.615	-1.1451	3.1807
	Group H	-0.89333	0.8339	0.707	-3.0563	1.2696
	Group ZH	-2.71111^*^	0.8339	0.007	-4.874	-0.5482
Group ZH	Group A	3.72889^*^	0.8339	0	1.566	5.8918
	Group H	1.81778	0.8339	0.133	-0.3451	3.9807
	Group ZA	2.71111^*^	0.8339	0.007	0.5482	4.8740

Table 4 shows multiple comparisons in terms of shrinkage made between Group A, Group H, Group ZA, and Group ZH, with mean differences of 24.578 mm^3^, 17.356 mm^3^, and 39.178 mm^3^, respectively. When Group H was compared with Groups ZA and ZH, the mean difference was -7.222 mm^3^ and 14.600 mm^3^. The mean difference was 21.822 mm^3^ when Groups ZA and ZH were compared. All the comparisons for polymerization shrinkage among the groups had a standard error of 1.05 and a p-value < 0.05.

**Table 4:** Multiple comparisons of polymerization shrinkage among the groups using Tukey’s HSD test * The mean difference is significant at the 0.05 level. HSD, honestly significant difference

(I) Group	(J) Group	Mean difference (I-J)	Standard error	p-value	95% CI	
					Lower bound	Upper bound
Group A	Group H	24.578^*^	1.051	0	21.85	27.3
	Group ZA	17.356^*^	1.051	0	14.63	20.08
	Group ZH	39.178^*^	1.051	0	36.45	41.9
Group H	Group A	-24.578^*^	1.051	0	-27.3	-21.85
	Group ZA	-7.222^*^	1.051	0	-9.95	-4.5
	Group ZH	14.600^*^	1.051	0	11.87	17.33
Group ZA	Group A	-17.356^*^	1.051	0	-20.08	-14.63
	Group H	7.222^*^	1.051	0	4.5	9.95
	Group ZH	21.822^*^	1.051	0	19.1	24.55
Group ZH	Group A	-39.178^*^	1.051	0	-41.9	-36.45
	Group H	-14.600^*^	1.051	0	-17.33	-11.87
	Group ZA	-21.822^*^	1.051	0	-24.55	-19.10

## Discussion

Newer high-strength dental ceramics are more effectively utilized when processed with CAD/CAM technology for creating fixed prostheses [7]. CAD/CAM technology results in the generation of powder waste, which should be researched for potential reuse [8]. Studies have stated that CAD/CAM waste can be used for sandblasting and that this residual powder can be considered a pigment [9,10]. Elagayar and Aboushelib in 2014 utilized the finer fraction (less than 40 μm), which was filtered directly from the CAD/CAM powder before being used to make fresh milling discs [11]. The present study has focused on recycling the zirconia dust collected after the subtractive process and reinforcing it in the poly(methyl methacrylate) (PMMA) matrix to improve its mechanical properties.

A study by Gouveia et al. on the microstructure and mechanical characterization of the residual zirconia powder concluded that the distribution of the particles was homogenous and uniform [2]. Ding et al. collected residual zirconia powder, upcycled it into blanks, and subjected them to various mechanical tests [1]. In the present study, one of the parameters under evaluation was microhardness. Ana et al. emphasized the significance of microhardness in provisional restorations, noting that the surface hardness of a material serves as an indicator of its ability to withstand wear and surface deterioration [12].

The study found significant variations in microhardness and polymerization shrinkage between autopolymerizing and heat-polymerized resin samples with and without zirconia dust. There was a significant difference in microhardness between heat polymerizing resin with zirconia and autopolymerizing resin with and without zirconia, with p-values of 0.07 and 0.00. Strong interfacial adhesion of the filler particles to the polymer matrix is essential for efficient stress transmission, which raises the hardness of the material. Additionally, the presence of zirconia particles may also hinder the propagation of cracks within the material, further contributing to its enhanced mechanical properties. The outcomes of the current study demonstrate that the addition of zirconia dust significantly enhances the microhardness of both autopolymerizing and heat-polymerized resins [13].

In a similar study, Ozdogan and Karslioglu explored the mechanical properties of a PMMA matrix incorporating varying proportions of residual Yttria-stabilized zirconia (YSZ) obtained from subtractive milling. The findings indicated that the inclusion of 40% wt of zirconia dust improved the microhardness of the PMMA matrix [13]. Alhareb and Ahmad examined the properties of heat-cured denture resin with the addition of 5 wt% of aluminum and zirconium oxide fillers and concluded that there was a significant increase in the tensile and flexural properties of the resin material [14]. This supports the notion that the addition of zirconium fillers can enhance the overall performance of acrylic resin in dental applications. Based on the results, it can be inferred that heat-polymerized resin exhibits higher microhardness compared to autopolymerizing resin. However, the addition of zirconia dust alone could not increase the hardness values beyond the values of heat-activated resins without YSZ. This shows that the polymerization method plays a significant role in the hardness of acrylic resins.

The second parameter assessed in this study was polymerization shrinkage. According to Libecki et al., dental resin polymerization shrinkage is attributed to two phenomena [15]. The first involves the shift from van der Waals distance between monomer molecules to a covalent bond during polymerization. The second occurs when the intermolecular distance between polymer chains becomes smaller than that between monomers. This shrinkage leads to the buildup of residual tensile stress around particles, interfacial bond failure, and microleakage, all of which have adverse effects on the material’s toughness. In the current study, shrinkage analysis revealed a significant mean difference of 24.57 ± 1.05 between autopolymerized and heat-polymerized samples, indicating reduced shrinkage due to the polymerization method. Comparing autopolymerized with zirconia dust to heat-polymerized with zirconia dust showed a mean difference of 21.82 ± 1.05, suggesting differing impacts of zirconia dust on each resin type. A larger mean difference of 39.17 ± 1.05 between pure autopolymerized samples and heat-polymerized samples with zirconia dust highlighted the substantial effect of both polymerization technique and zirconia addition on shrinkage.

Balkenhol et al. stated that polymerization shrinkage induces dimensional alterations in the temporary restoration, resulting in compromised precision of fit and the development of internal stresses within the restoration [16]. In the current study, the shrinkage was significantly reduced with the addition of zirconia dust to autopolymerized and heat-polymerized resin. Vallittu et al. stated that there are higher amounts of residual monomers in autopolymerizing resin than in heat-cured resin. Furthermore, it was observed that autopolymerized samples with zirconia dust had more shrinkage than heat-polymerized samples with zirconia dust [17]. This difference in shrinkage depended on the type of polymerization process, i.e., heat curing, and also on the addition of residual zirconia. These findings highlight the complexities and significant effects that both the addition of zirconia dust and the choice of polymerization method have on the shrinkage property of dental resins.

The limitations of the study were that the experiments were conducted under controlled laboratory conditions, which might not fully replicate the complex conditions in oral environments. Various types of polymerization shrinkages were not factored in, and properties such as wear resistance, color stability, and the long-term impact of oral fluids on material integrity may not have been fully assessed. Addressing these limitations in future research could help refine the understanding of how zirconia dust incorporation affects dental resins and expand the potential applications of these materials in dentistry. Future research could focus on investigating different concentrations of zirconia dust, and various types of resins and fillers could identify optimal formulations. Ensuring and verifying the uniform distribution of zirconia dust within the resin matrix, analyzing the cost and manufacturing feasibility, and conducting clinical trials to validate laboratory findings in real-world settings are crucial steps. Additionally, aging and degradation studies will be important to ensure the longevity and stability of residual zirconia-incorporated resins in dental restorations. This multifaceted approach will deepen insights into the benefits and potential limitations of residual zirconia-incorporated resins, leading to more advanced and reliable materials for provisional crowns and other dental applications.

## Conclusions

The initial research into the incorporation of zirconia dust in dental resins has provided a foundational understanding of its potential benefits. Within the limitations of the study, it can be concluded that the highest microhardness and lowest polymerization shrinkage were observed in heat-polymerized resin samples containing residual zirconia. The incorporation of zirconia dust in both autopolymerized and heat-polymerized resin samples enhanced the microhardness. Pure heat-polymerized resin had more hardness than autopolymerized resin incorporated with zirconia dust. There was a significant decrease in the shrinkage values in the heat- and autpolymerized samples after the incorporation of residual zirconia. It can be concluded that the addition of zirconia dust to provisional crowns can be one of the potential ways to recycle the CAD/CAM residual waste.
